# Visualization of Calcium Ion Loss from Rotavirus during Cell Entry

**DOI:** 10.1128/JVI.01327-18

**Published:** 2018-11-27

**Authors:** Eric N. Salgado, Brian Garcia Rodriguez, Nagarjun Narayanaswamy, Yamuna Krishnan, Stephen C. Harrison

**Affiliations:** a Laboratory of Molecular Medicine, Boston Children’s Hospital, Harvard Medical School, Boston, Massachusetts, USA; b Howard Hughes Medical Institute, Boston, Massachusetts, USA; c Department of Chemistry, University of Chicago, Chicago, Illinois, USA; d Grossman Institute of Neuroscience, Quantitative Biology and Human Behavior, University of Chicago, Chicago, Illinois, USA; University of Pittsburgh School of Medicine

**Keywords:** Ca^2+^ sensor, live-cell imaging, membrane penetration, rotavirus entry

## Abstract

Bound calcium ions stabilize many nonenveloped virions. Loss of Ca^2+^ from these particles appears to be a regulated part of entry or uncoating. The outer layer of an infectious rotavirus triple-layered particle (TLP) comprises a membrane-interacting protein (VP4) anchored by a Ca^2+^-stabilized protein (VP7). Membrane-coupled conformational changes in VP4 (cleaved to VP8* and VP5*) and dissociation of VP4 and VP7 accompany penetration of the double-layered inner capsid particle (DLP) into the cytosol. Removal of Ca^2+^ *in vitro* strips away both outer layer proteins; we and others have postulated that the loss of Ca^2+^ triggers molecular events in viral penetration. We have now investigated, with the aid of a fluorescent Ca^2+^ sensor, the timing of Ca^2+^ loss from entering virions with respect to the dissociation of VP4 and VP7. In live-cell imaging experiments, distinct fluorescent markers on the DLP and on VP7 report on outer layer dissociation and DLP release. The Ca^2+^ sensor, placed on VP5*, monitors the Ca^2+^ concentration within the membrane-bound vesicle enclosing the entering particle. Slow (1-min duration) loss of Ca^2+^ precedes the onset of VP7 dissociation by about 2 min and DLP release by about 7 min. Coupled with our previous results showing that VP7 loss follows tight binding to the cell surface by about 5 min, these data indicate that Ca^2+^ loss begins as soon as the particle has become fully engulfed within the uptake vesicle. We discuss the implications of these findings for the molecular mechanism of membrane disruption during viral entry.

**IMPORTANCE** Nonenveloped viruses penetrate into the cytosol of the cells that they infect by disrupting the membrane of an intracellular compartment. The molecular mechanisms of membrane disruption remain largely undefined. Functional reconstitution of infectious rotavirus particles (TLPs) from RNA-containing core particles (DLPs) and the outer layer proteins that deliver them into a cell makes these important pediatric pathogens particularly good models for studying nonenveloped virus entry. We report here how the use of a fluorescent Ca^2+^ sensor, covalently linked to one of the viral proteins, allows us to establish, using live-cell imaging, the timing of Ca^2+^ loss from an entering particle and other molecular events in the entry pathway. Specific Ca^2+^ binding stabilizes many other viruses of eukaryotes, and Ca^2+^ loss appears to be a trigger for steps in penetration or uncoating. The experimental design that we describe may be useful for studying entry of other viral pathogens.

## Abstract (precis)

Nonenveloped viruses penetrate into the cytosol of the cells that they infect by disrupting the membrane of an intracellular compartment. The molecular mechanisms of membrane disruption remain largely undefined. Functional reconstitution of infectious rotavirus particles (TLPs) from RNA-containing core particles (DLPs) and the outer layer proteins that deliver them into a cell makes these important pediatric pathogens particularly good models for studying nonenveloped virus entry. We report here how the use of a fluorescent Ca^2+^ sensor, covalently linked to one of the viral proteins, allows us to establish, using live-cell imaging, the timing of Ca^2+^ loss from an entering particle and other molecular events in the entry pathway. Specific Ca^2+^ binding stabilizes many other viruses of eukaryotes, and Ca^2+^ loss appears to be a trigger for steps in penetration or uncoating. The experimental design that we describe may be useful for studying entry of other viral pathogens.

## INTRODUCTION

The 10,000-fold difference between the concentration of Ca^2+^ in the cytosol and its concentration in the extracellular environment is among the most important cellular markers of “in” and “out” (1). It permits great flexibility in the intensity, timing, and localization of changes in Ca^2+^ concentrations and hence considerable subtlety in Ca^2+^-based signaling mechanisms. It also permits entering molecules to detect when they have crossed a cellular membrane, either from the cell surface or from an internal, membrane-bound compartment, and arrived in the cytosol.

**FIG 1:**
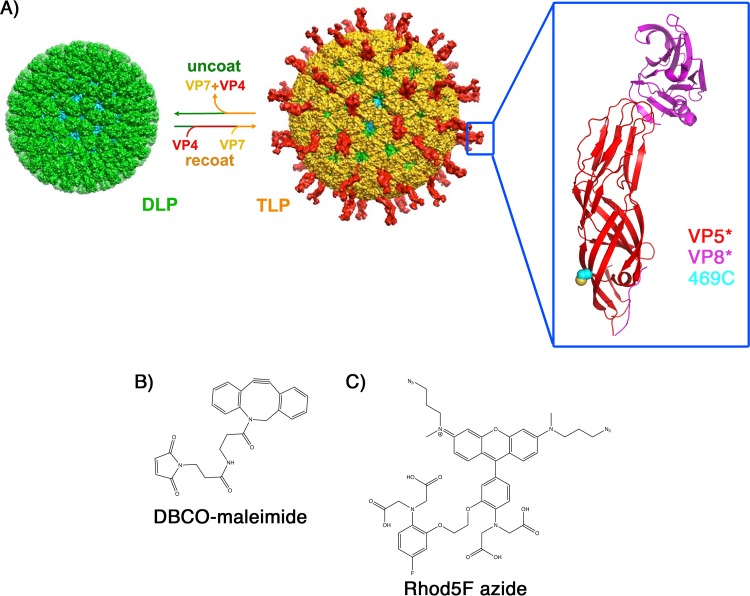
Rotavirus structure, reconstitution, location of the S469C mutation in VP4, and structures of the Ca^2+^ sensor components. (A) Rotavirus structural components, including the outer coat proteins VP4 (red) and VP7 (yellow) and the DLP proteins VP6 (green) and VP2 (cyan). The double-layer particle (DLP) of rotavirus can be reconstituted into an infectious triple-layer particle (TLP) by the addition of VP4 and VP7 in the presence of Ca^2+^ (19). Chelating Ca^2+^ ions dissociates VP7 trimers, stripping off the outer layer of the TLP. The blue box indicates the VP4 spike, magnified in the larger box, showing the location of the S469C mutation (yellow and cyan spheres) and the VP5* “body” and VP8* “lectin” subdomains of VP4 (the “foot” subdomain of VP8* plus VP5* is not shown [see reference 8]). (B) Structure of DBCO-maleimide. (C) Structure of Rhod5F azide.

Various nonenveloped viruses have evolved to depend on bound Ca^2+^ for particle stability and hence to respond to its loss by conformational changes that lead to uncoating or disassembling. Examples include the polyomaviruses, most T = 3 plant viruses, and rotaviruses (2–7). The infectious form of rotavirus, the so-called triple-layered particle (TLP) (Fig. 1A), contains an icosahedral (T = 13) double-layered particle (DLP) core, surrounded by a shell of viral protein 7 (VP7) trimers. The VP7 shell anchors 60 trimeric VP4 spikes onto the particle surface (8). Ca^2+^ ions bound specifically at the subunit interfaces hold the VP7 trimers together (9). Chelating these Ca^2+^ ions strips away both VP7 and VP4, leaving an intact DLP. A related process occurs during cell entry. The TLP binds ganglioside receptors on the cell membrane (10, 11) and generates, by invagination and budding, a tightly fitting vesicle (12). Progressive loss of VP7 and VP4 leads, after several minutes, to the release of the DLP into the cytosol. The transcriptionally active DLP contains the 11 segments of the viral double-stranded RNA (dsRNA) genome, each associated with a copy of the viral polymerase VP1. DLP release is thus the critical step for initiating infection (13–16).

Various experimental approaches have shown that VP4 is the key agent of vesicle-membrane disruption and DLP release. Proteolytic cleavage to VP8* and VP5* activates VP4 (17, 18), enabling VP5* to undergo membrane-linked conformational changes (19–21). In previous work, we monitored the entry events outlined above by generating reconstituted TLPs (rcTLPs) from DLPs and recombinant VP7 and VP4 (12, 22). Distinct fluorescent markers on the DLP and on VP7 allowed us to monitor directly, by the use of live-cell imaging, the loss of VP7 and release of the DLP. Installation of a third fluorophore on VP4 can then permit us to monitor the timing of other features of the entry process, with respect to VP7 uncoating and DLP release. We have now taken advantage of this scheme to monitor the local drop in Ca^2+^ concentrations by using a conjugatable Ca^2+^ sensor, rhodamine 5F (Rhod5F) azide (Fig. 1C) (23). We generated a mutant VP4 with a cysteine at a position unlikely to affect the stability of known conformers of VP5* and developed a scheme for installing Rhod5F at that position. We show here that there is a relatively slow (ca. 1-min) loss of Ca^2+^ from the vesicular compartment surrounding a virion. This event precedes VP7 decapsidation by about 2 min and DLP release by about 7 min. The results constrain models for the molecular mechanism of membrane disruption and illustrate the utility of directly monitoring Ca^2+^ levels during uptake of macromolecules into cells.

## RESULTS

### Reconstitution and imaging of Atto 565-labeled rcTLPs.

**FIG 2:**
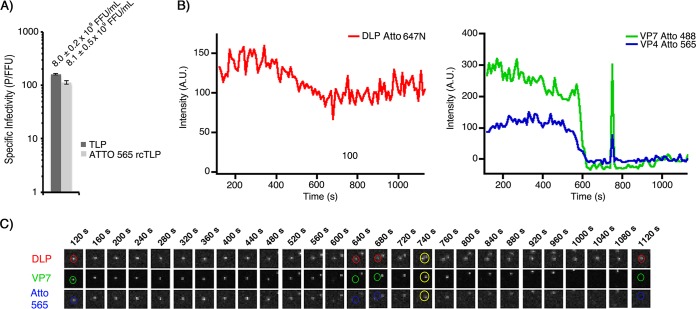
Infectivity and live-cell imaging of Atto 565 rcTLPs. (A) Focus-forming assay comparing infectivities of native RRV (TLP) with Atto 565-labeled rcTLPs. rcTLPs were generated from DLPs, VP7, and VP4 labeled with Atto 647N, Atto 488, and Atto 565 dyes, respectively. Specific infectivity is shown as particles per focus-forming unit from triplicate experiments in BSC-1 cells. Error bars indicate standard deviations (SD). Infectivity and SD (focus-forming units per milliliter) of virus in each sample are shown above the respective bars. (B) BSC-1 cells were infected at an MOI of 50 with Atto 565 rcTLPs and imaged for 30 min. Individual particles were tracked by the signal in the 642-nm channel (DLP) (see Materials and Methods); uncoating events were detected as particles with a roughly constant 642-nm signal that lose the 488-nm signal (VP7). A similar loss in the 561-nm channel (VP4) coincides with the loss of VP7. The trace is representative of hundreds of measurements: another example is shown in Fig. 3. The spike in the 488- and 561-nm traces at about 740 s (see the 740-s time point in panel C), well after the particle in question has lost intensity in these channels, comes from the transient proximity of a second rcTLP that has not uncoated and thus has a fluorescent signal in both channels. These increased signals disappear when the two particles move away from one another in subsequent images. (C) Micrographs from all three channels of the rcTLP represented in panel B. Circular outlines highlight the particle in question in the 642-nm (DLP), 488-nm (VP7), and 561-nm (VP4) channels. A yellow outline in the 740-s frame shows a second rcTLP, which approaches the particle being monitored, causing the transient spikes in the 488- and 561-nm fluorescence intensity traces in panel B. A.U., arbitrary units.

We prepared wild-type (wt) recombinant VP4 (rVP4) as described previously (22) and labeled it with Atto 565 *N*-hydroxysuccinamide (NHS) ester (VP4-565) at a ratio of ∼1 dye molecule per protein monomer. Atto 647N NHS ester-labeled DLPs (DLP-647N) were then recoated with VP4-565 and Atto 488 NHS ester-labeled VP7 (VP7-488), as described in Materials and Methods. The specific infectivity of these Atto 565 rcTLPs was 113 particles per focus-forming unit (P/FFU) (Fig. 2A), slightly higher than the 161 P/FFU for the native TLP sample tested on the same day, a relationship that we have found previously (16).

Atto 565 rcTLPs were added to BSC-1 cells at a multiplicity of infection (MOI) of 50 and imaged for 30 min, as described in Materials and Methods. Tracking of the labeled DLPs by their intensity in the 642-nm channel yielded 127 total uncoating events (Fig. 2B and C). As in previous work (12), we defined uncoating as a particle trajectory in which the VP7 intensity (488-nm channel) was reduced to background levels while the DLP intensity remained roughly constant. After uncoating, we monitored the DLP until the particle began to move rapidly away from the site of uncoating (“release”).

**FIG 3:**
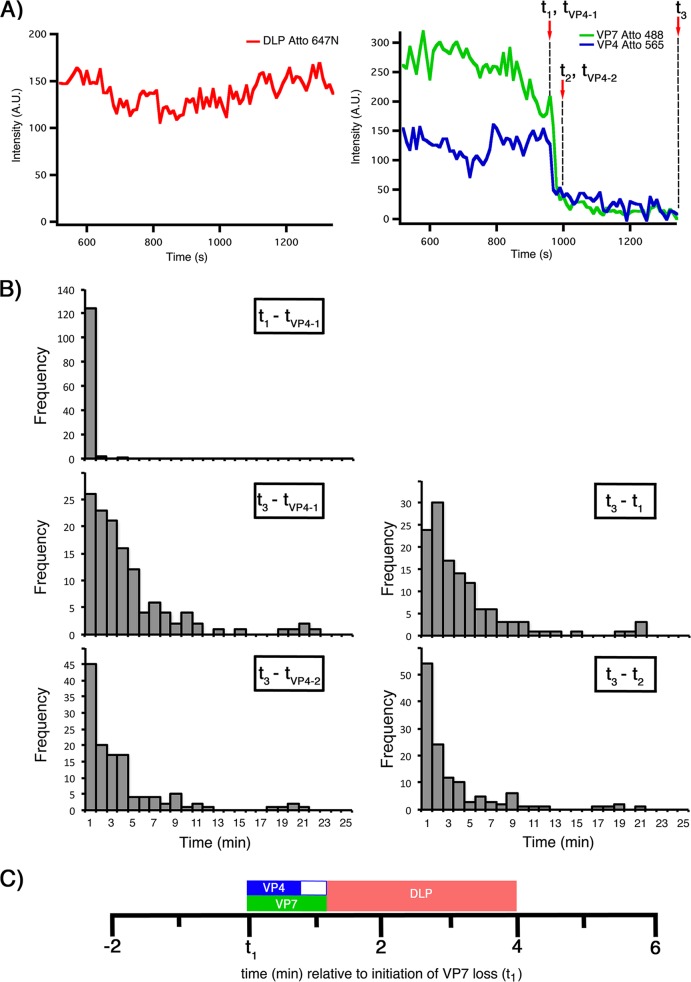
Kinetics of VP7 and VP4 loss and of DLP release from Atto 565 rcTLPs. (A) Traces for DLP (left) and VP7 and VP4 (right). Dashed vertical lines show the onset of VP7 loss (*t*_1_) and termination of VP7 loss (*t*_2_), the onset of VP4 loss (*t*_VP4-1_) and its termination (*t*_VP4-2_), and DLP release (*t*_3_). See the legend of Fig. 1B for details. (B) Distribution of time intervals between the onset of VP7 loss and VP4 loss (*t*_1_ − *t*_VP4-1_), the onset of VP4 loss and DLP release (*t*_3_ − *t*_VP4-1_), the onset of VP7 loss and DLP release (*t*_3_ − *t*_1_), termination of VP4 loss and DLP release (*t*_3_ − *t*_VP4-2_), and termination of VP7 loss and DLP release (*t*_3_ − *t*_2_) from 127 uncoating particles collected from 7 BSC-1 cells infected at an MOI of 50. (C) Schematic of the average times outlined in panel B demonstrating the timing and duration of VP7 loss (green bar), VP4 loss (blue bar), and overall DLP release (red bar), in relation to *t*_1_.

Analysis of these data showed that initiation of VP7 signal loss in the 488-nm channel (*t*_1_) (Fig. 3) coincided with initiation of VP4 signal loss in the 560-nm channel (*t*_VP4-1_) (Fig. 3B and C and Table 1). The average interval between *t*_1_ or *t*_VP4-1_ and subsequent DLP release (*t*_3_) was 4.0 ± 0.4 min. The average interval (*t*_3_ − *t*_2_) between the endpoint of VP7 loss (*t*_2_) and release (*t*_3_) was 2.9 ± 0.4 min.

**TABLE 1:** Kinetics of VP7 and VP4 loss, and DLP release, from Atto 565 rcTLPs^*a*^

Interval	Mean avg time (min) ± SD	Median time (min)
*t*_1_ − *t*_VP4-1_	0.04 + 0.05	0.0
*t*_3_ − *t*_1_	4.0 ± 0.4	2.5
*t*_3_ − *t*_VP4-1_	4.0 ± 0.4	2.7
*t*_3_ − *t*_2_	2.9 ± 0.4	1.5
*t*_3_ − *t*_VP4-2_	3.3 ± 0.4	2.0

a Number of particles, 127; number of cells, 7.

### Reconstitution and imaging of pHrodo Red-labeled rcTLPs.

**FIG 4:**
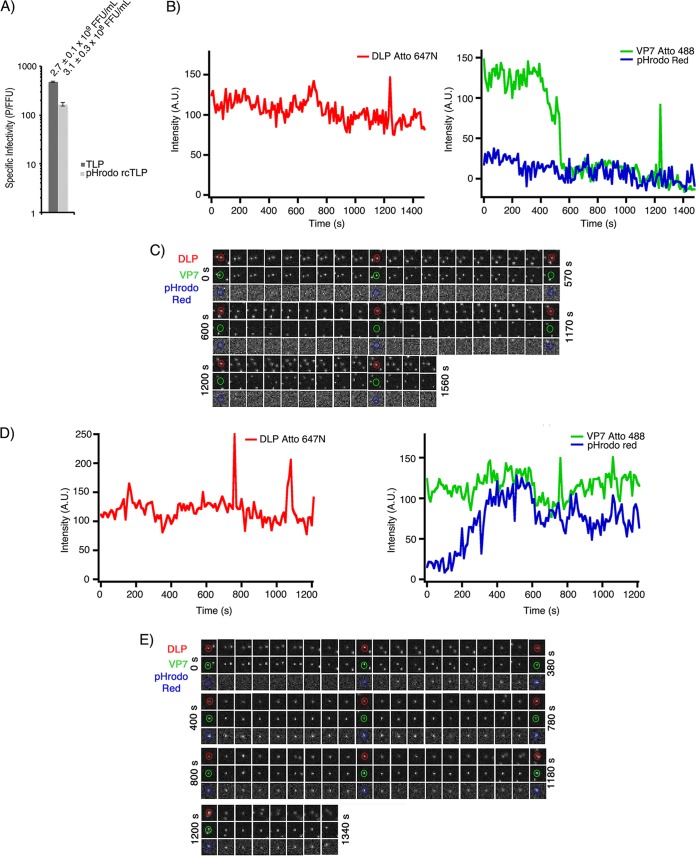
pHrodo Red rcTLPs. (A) Focus-forming assay comparing infectivities of native RRV (TLP) and pHrodo Red-labeled rcTLPs. rcTLPs generated from DLPs, VP7, and VP4 were labeled with Atto 647N, Atto 488, and pHrodo Red dyes, respectively. Specific infectivities are shown as particles per focus-forming unit from triplicate experiments in BSC-1 cells. Error bars indicate SD. Infectivity and SD (focus-forming units per milliliter) of the virus in each sample are shown above the respective bars. (B) BSC-1 cells were infected at an MOI of 50 with pHrodo Red rcTLPs and imaged for 30 min. Individual particles were tracked by the signal in the 642-nm channel (red trace) (see Materials and Methods). Uncoating events were detected as particles with a roughly constant 642-nm signal that lose the 488-nm signal (VP7). Uncoating occurred in the absence of any increase in 561-nm intensity (VP4). A trace of a different particle from the same experiment is shown in Fig. 7A. (C) Micrographs from all three channels of the rcTLP represented in panel B. Circular outlines highlight the particle in question in the 642-nm (DLP), 488-nm (VP7), and 561-nm (VP4) channels. The time interval between frames was 30 s. (D) Particles that failed to uncoat during the time of data collection had constant signals in both the 642- and 488-nm channels. These particles eventually colocalized with increased signals in the pHrodo Red (561-nm) channel (i.e., a pH drop). (E) Micrographs from all three channels of the rcTLP represented in panel D. Circular outlines highlight the particle in question in the 642-nm (DLP), 488-nm (VP7), and 561-nm (VP4) channels. The time interval between frames was 20 s.

We labeled wild-type VP4 with pHrodo Red (Invitrogen) succinimidyl ester (VP4-pHrodo Red) at a ratio of 1 dye molecule per 2 VP4 monomers and recoated DLP-647N with VP4-pHrodo Red and VP7-488, as described in Materials and Methods. The specific infectivity of these pHrodo Red rcTLPs was 165 P/FFU (Fig. 4A), which was again higher than that of native TLPs tested on the same day with a specific infectivity of 477 P/FFU.

**FIG 5:**
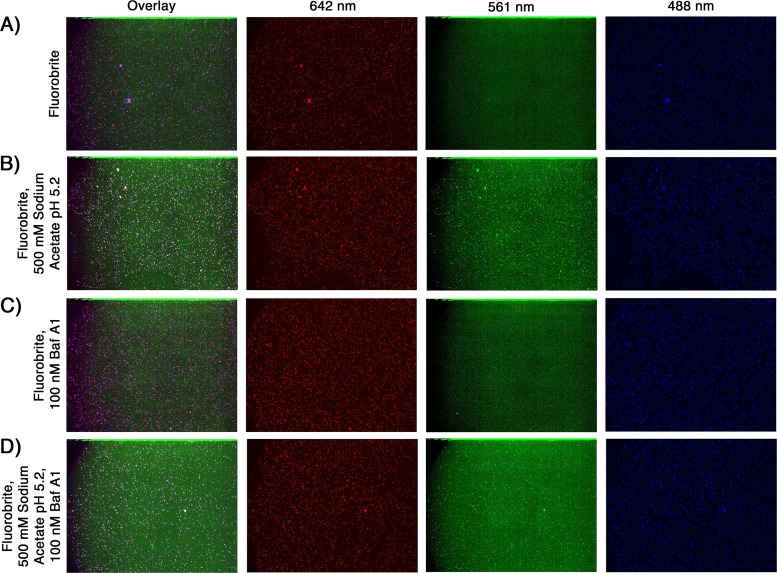
Response to reduced pH of pHrodo Red rcTLPs on glass coverslips. pHrodo Red rcTLPs with DLP-647N, VP7-488, and VP4-pHrodo Red were imaged in the same Fluorobrite medium used for live-cell imaging (see Materials and Methods). Each field of particles was imaged at wavelengths of 642 nm (red), 562 nm (green), and 488 nm (blue) in a 15-step z-series (0.5-μm step size). Shown are maximum-intensity z-projections of an overlay of all three channels as well as individual channels. (A) Fluorobrite medium; (B) Fluorobrite medium supplemented with 500 mM sodium acetate (pH 5.2); (C) Fluorobrite medium with 1% DMSO and 100 nM Baf A1; (D) Fluorobrite medium with 1% DMSO and 100 nM Baf A1 supplemented with 500 mM sodium acetate (pH 5.2).

We verified the response to changes in pH of pHrodo Red-labeled rcTLPs by imaging the reconstituted particles on glass in Fluorobrite Dulbecco’s modified Eagle’s medium (DMEM). The pHrodo Red rcTLPs emitted no fluorescent signal in the 562-nm channel in medium alone (Fig. 5A), but the addition of sodium acetate (pH 5.2) to the sample gave rise to a signal in the 562-nm channel that colocalized with both the DLP (640-nm) and VP7 (488-nm) signals of individual particles (Fig. 5B).

**FIG 6:**
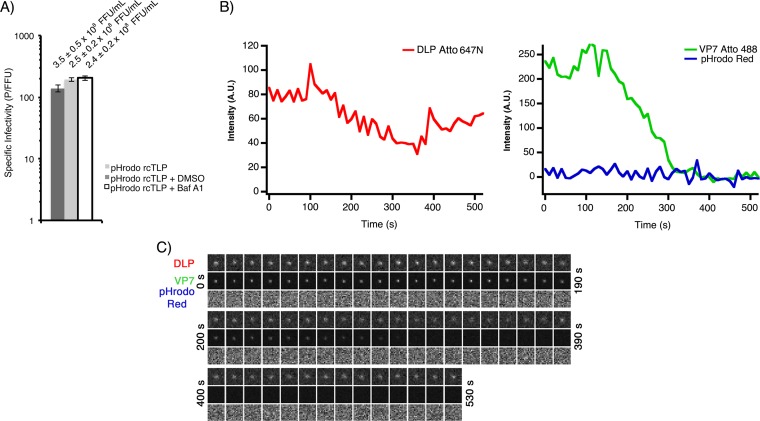
Infectivity and live-cell imaging of pHrodo Red rcTLPs in the presence of Baf A1. (A) Focus-forming assay comparing infectivities of pHrodo Red-labeled rcTLPs infecting BSC-1 cells in DMEM, DMEM with 100 nM Baf A1 and 1% DMSO, or 1% DMSO in DMEM (see Materials and Methods). Specific infectivity is shown as particles per focus-forming unit from triplicate experiments in BSC-1 cells. Error bars indicate SD. Infectivity and SD (focus-forming units per milliliter) are shown above the respective bars. (B) BSC-1 cells were incubated in medium containing 100 nM Baf A1 and 1% DMSO for 1 h at 37°C before infection at an MOI of 50 with pHrodo Red rcTLPs and imaging for 30 min. Individual particles were tracked by the signal in the 642-nm channel (red trace) (see Materials and Methods). Uncoating events were detected as particles with a roughly constant 642-nm signal that lose the 488-nm signal (VP7). Uncoating occurred in the absence of any increase in the 561-nm intensity (VP4). (C) Micrographs of all three channels of the rcTLP represented in panel B. The time interval between frames was 10 s.

pHrodo Red rcTLPs were added to BSC-1 cells at an MOI of 50 and imaged for 30 min, as described in Materials and Methods. Tracking of the labeled DLPs by their intensity in the 642-nm channel yielded 253 total uncoating events, none of which was accompanied by the appearance of a signal in the 561-nm channel (Fig. 4B and C). Those particles that emitted a signal in the pHrodo Red channel had not uncoated, even at the end of the acquisition time (Fig. 4D and E). Treatment of cells with bafilomycin A1 (Baf A1), a known inhibitor of the vacuolar-type H^+^-ATPases, did not change the infectivity of the pHrodo Red rcTLP (Fig. 6A), nor did Baf A1 have any effect on the pH response of the labeled rcTLPs on glass (Fig. 5C and D). These observations are consistent with our previous studies showing that rhesus rotavirus (RRV) uncoats before it reaches an acidic endosome and that particles reaching such endosomes never uncoat (12).

Live-cell imaging of Baf A1-treated BSC-1 cells infected with pHrodo Red-labeled rcTLPs at an MOI of 50 yielded 81 uncoating events. As in Baf A1-free samples, particles that uncoated did so in the absence of a colocalized 561-nm signal (Fig. 6B and C). No particles that remained as intact rcTLPs developed a 561-nm signal in these experiments (data not shown), consistent with the inhibition of vacuolar-type H^+^-ATPases.

**FIG 7:**
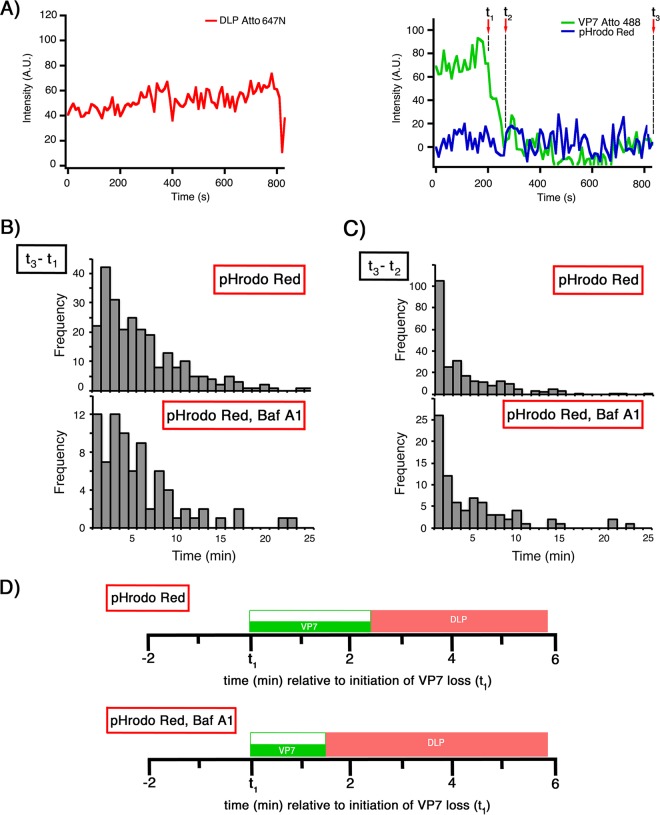
Kinetics of VP7 loss and DLP release from pHrodo Red rcTLPs. (A) Traces for DLP (left) and for VP7 and pHrodo Red (right). Dashed vertical lines show the onset of VP7 loss (*t*_1_), termination of VP7 loss (*t*_2_), and DLP release (*t*_3_). See the legend of Fig. 1B for details. (B) Distribution of intervals between the onset of VP7 loss and DLP release (*t*_3_ − *t*_1_) in BSC-1 cells infected in the absence (top) or presence (bottom) of Baf A1. (C) Distribution of times between the termination of VP7 loss and DLP release (*t*_3_ − *t*_1_) in BSC-1 cells infected in the absence (top) or presence (bottom) of Baf A1. (D) Schematics of the average times outlined in panel B demonstrating the timing and duration of VP7 loss (green bars) and overall DLP release (red bars) in relation to *t*_1_ in the absence (top) or presence (bottom) of Baf A1.

The times between the initiation of VP7 loss and DLP escape (*t*_3_ − *t*_1_) are 5.9 ± 0.3 min and 5.8 ± 0.6 min in the absence and presence of Baf A1, respectively (Fig. 7 and Table 2). The *t*_3_ − *t*_2_ intervals for these data were 3.4 ± 0.3 min and 4.4 ± 0.6 min, respectively.

**TABLE 2:** Kinetics of VP7 loss and DLP release from pHrodo Red rcTLPs

Interval	Avg time (min) ± SD	Median time (min)	No. of particles	No. of cells
*t*_3_ − *t*_1_				
pHrodo Red	5.9 ± 0.3	4.4	253	10
pHrodo Red, Baf A1	5.8 ± 0.6	4.0	81	5
*t*_3_ − *t*_2_				
pHrodo Red	3.4 ± 0.3	2.0		
pHrodo Red, Baf A1	4.4 ± 0.6	2.7		

### Rhod5F labeling of VP4 469C and formation of VP5 CT digestion fragments.

**FIG 8:**
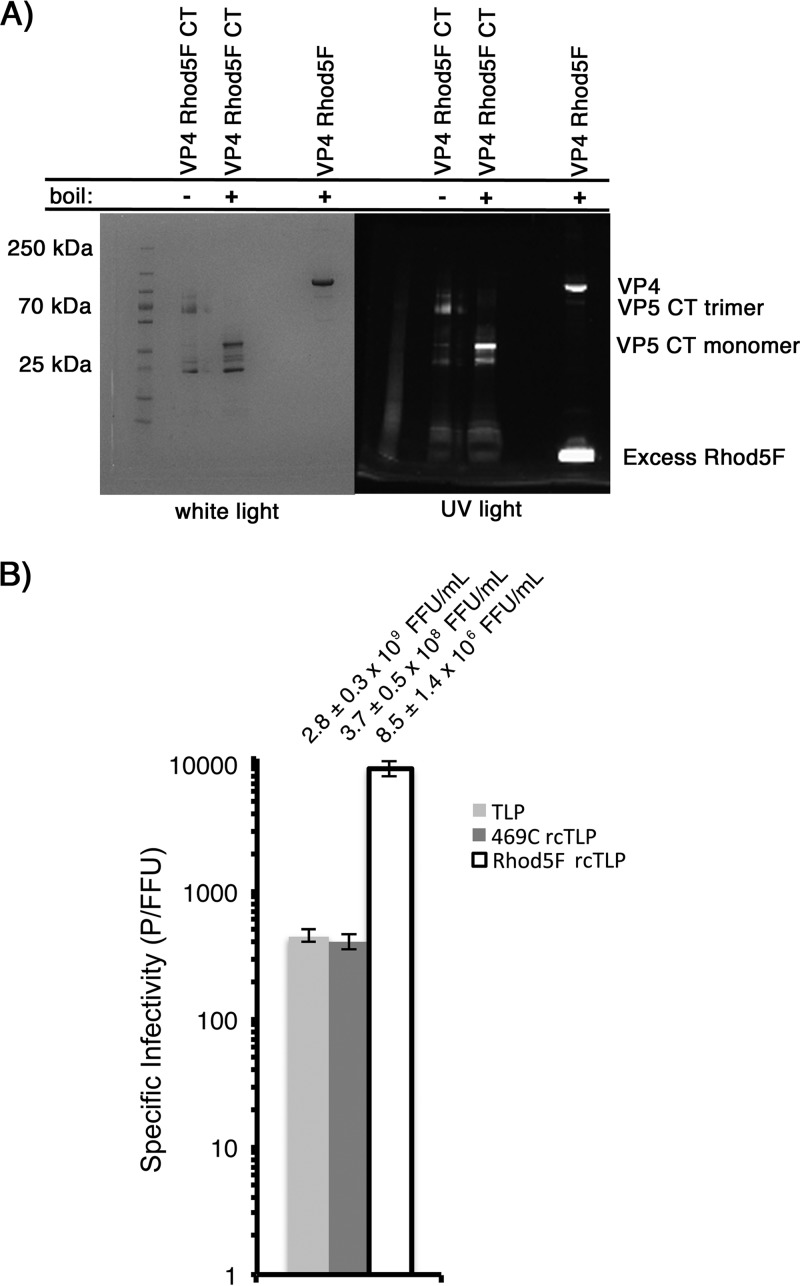
VP5 CT digestion of VP4 Rhod5F and infectivity of Rhod5F rcTLPs. (A) Monomeric VP4 469C was sequentially labeled with DBCO-maleimide and Rhod5F azide as described in Materials and Methods. The resulting labeled VP4 Rhod5F was sequentially digested with chymotrypsin and trypsin (see Materials and Methods). Reducing sample buffer was added to a sample of undigested VP4 Rhod5F that was then boiled and subjected to analysis by SDS-PAGE. Reducing sample buffer was also added to two samples of the digested protein, one of which was boiled prior to SDS-PAGE; the other sample remained unboiled. Samples were then run on a 5-to-20% gradient SDS gel and imaged with UV light (right) prior to staining with Coomassie blue and imaging with transmitted light (left). (B) Focus-forming assay comparing infectivities of native RRV (TLP), 469C rcTLPs, and Rhod5F rcTLPs (see Materials and Methods). Specific infectivity is shown as particles per focus-forming unit from triplicate experiments in BSC-1 cells. Error bars indicate SD. Infectivity ± SD (focus-forming units per milliliter) for the virus in each sample are shown above the respective bars.

We expressed VP4 mutated at position 469 from serine to cysteine (VP4 469C) and labeled the protein sequentially with dibenzocyclooctyne-maleimide (DBCO-maleimide) and Rhod5F azide (Fig. 8A; see also Materials and Methods) at a final ratio of 1 Rhod5F molecule to each VP4. We refer to the product as VP4 Rhod5F.

The relatively large combined size of the DBCO linker and Rhod5F azide might in principle hinder the VP4 conformational change proposed to be necessary for cell entry. The final, folded-back conformation is represented by the structure of the sequentially chymotrypsin-trypsin (CT)-digested VP5 CT fragment (21). We therefore subjected VP4 Rhod5F to sequential chymotrypsin and trypsin digestion. Apparent molecular masses (from SDS-PAGE) of the VP5 CT fragment were approximately 60 kDa for a reduced but unboiled trimer of roughly 60 kDa and about 30 kDa for a reduced and boiled monomeric form. Digestion of VP4 Rhod5F yielded essentially the same fragments under these conditions, with the full-length protein, digested trimer, and digested monomer all containing the fluorescent Rhod5F label (Fig. 8A).

### Infectivity and binding of Rhod5F-labeled rcTLPs.

Because VP4 is the limiting reagent in rcTLP reconstitution (22), we generated Rhod5F-labeled rcTLPs by first producing particles with unlabeled VP4 469C, VP7-488, and DLP-647N, purifying the reconstituted particles on a CsCl gradient to eliminate excess VP4 and VP7, and then labeling these 469C rcTLPs sequentially with DBCO-maleimide and Rhod5F azide. This procedure allowed us to compare the infectivities of the rcTLPs with and without the Rhod5F label from the same preparation. As shown in Fig. 8B, the 469C rcTLPs had a specific infectivity (407 P/FFU) similar to that of native TLPs (452 P/FFU). After labeling with the Ca^2+^ sensor, the Rhod5F rcTLPs had a specific infectivity of 9,290 P/FFU, roughly a 20-fold increase (Fig. 8B). We also observed reduced infectivity when we labeled VP4 469C Rhod5F before rcTLP reconstitution (data not shown). The bulky Rhod5F modification, which includes both the DBCO linker and the Ca^2+^ indicator itself, together with its proximity to the receptor binding domain VP8*, probably results in steric interference with the sialylated viral receptors on the surface of the cell, thus reducing the likelihood of binding by the Rhod5F rcTLPs.

**FIG 9:**
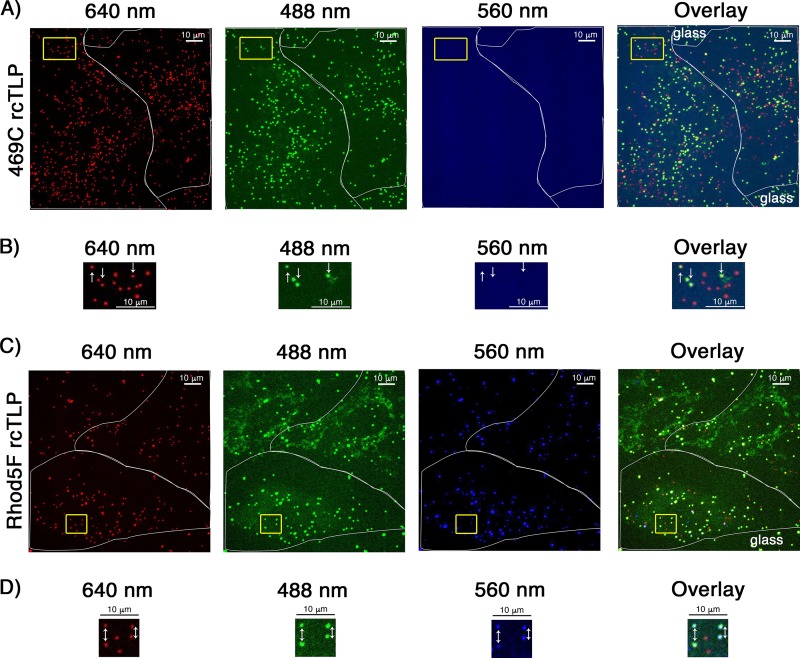
Cell binding by 469C and Rhod5F rcTLP. (A) BSC-1 cells were infected with rcTLPs with DLP-647N, VP7-488, and VP4 469C at an MOI of 10 for 1 h at 37°C (see Materials and Methods); washed in HNC; fixed for 10 min in 4% paraformaldehyde in HNC; and washed three more times for 5 min in HNC. The full volumes of 124 cells were imaged at 488, 561, and 642 nm as a z-series with a step size of 0.5 μm. Shown are representative maximum-intensity z-projections of micrographs of all three channels, pseudocolored red (642 nm), green (488 nm), and blue (561 nm), as well as an overlay of all three. The white outline shows the outer boundary of the cell; the yellow box highlights particles of interest on the cell. (B) Magnified image of particles highlighted in panel A and overlay of the three. White arrows indicate rcTLPs that have yet to lose the VP7-488 shell, showing that the unlabeled 469C rcTLPs lack the 560-nm Rhod5F signal. Other particles in the field of view represent uncoated DLP-647N particles. (C) BSC-1 cells were infected at an MOI of 1 for 1 h at 37°C with rcTLPs from the preparation used for images in panels A and B, sequentially labeled with DBCO-maleimide and Rhod5F azide (see Materials and Methods). The cells were then washed, fixed, and imaged as described above for panel A. Shown are representative z-projections from all three channels, pseudocolored red (642 nm), green (488 nm), and blue (561 nm), as well as an overlay of all three. The white outline highlights the outer boundary of the cell; the yellow box highlights particles of interest. (D) Magnified image of the particles highlighted in panel A by the yellow box in all three channels of interest and overlay of the three. White arrows point to rcTLPs, which have yet to lose the VP7-488 shell, showing that the Rhod5F rcTLPs colocalize with a Rhod5F signal in the 560-nm channel. All other particles in the field of view represent uncoated DLP-647N particles.

To define the source of this reduced infectivity, we infected BSC-1 cells at a viral load of about 10,000 particles per cell with Rhod5F rcTLPs (MOI = 1) and VP4 469C rcTLPs (MOI = 20). After a 1-h incubation, cells were washed to remove unbound particles, fixed, and observed by spinning-disk confocal fluorescence microscopy. Of the 10,000 VP4 469C rcTLPs added per cell, an average of 204 particles bound, corresponding to about 2% of the added particles (Fig. 9A and B and Table 3). An average of 94 of the bound particles, about 46%, lacked a signal in the 488-nm channel and had thus uncoated (see Materials and Methods). An average of 78 Rhod5F rcTLPs had bound per cell (Fig. 9C and D and Table 3), corresponding to 0.78% of the added particles; of these, an average of 26 (33%) had uncoated. The loss of infectivity upon labeling with Rhod5F is thus a result of poor cell attachment; once particles have attached, efficient entry and uncoating appear to be unaffected.

**TABLE 3:** Results of 469C and Rhod5F rcTLP binding assays

Label	No. of cells	Total no. of particles bound	Total no. of uncoated particles	% uncoated particles	Avg no. of particles bound/cell	Avg no. of uncoated particles/cell
469C	124	25,340	11,700	46	204	94
Rhod5F	120	9,341	3,080	33	78	26

### Live-cell imaging of Rhod5F-labeled rcTLPs.

**FIG 10:**
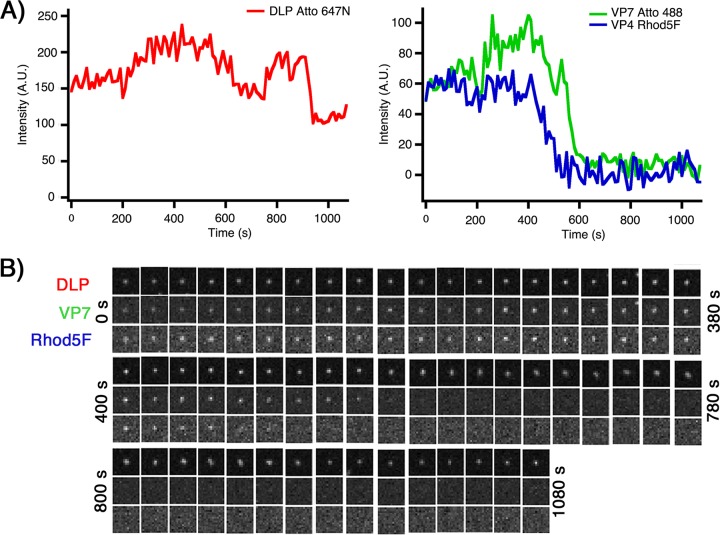
Live-cell imaging of Rhod5F rcTLPs. (A) BSC-1 cells were infected at an MOI of 10 with Rhod5F rcTLPs and imaged for 30 min. Individual particles were tracked by the signal in the 642-nm channel (left) (see Materials and Methods). Uncoating events were detected as particles with a roughly constant 642-nm signal that lose the 488-nm signal (VP7) (right). A decrease in the 561-nm intensity (VP4 Rhod5F) (right) preceded the onset of VP7 uncoating. Another example from the same experiment is shown in Fig. 9A. (B) Micrographs of all three channels of the uncoating rcTLP represented in panel A.

We infected BSC-1 cells at an MOI of 10 with Rhod5F rcTLPs and imaged them for 30 min. We tracked the DLP signal in the 642-nm channel, recording 222 uncoating events in 35 cells. Despite sufficient signals for both VP7 and DLP to track particles and classify uncoating events, the relatively weak fluorescence intensity of Rhod5F caused the signal to fall below noise if a particle moved slightly out of the plane of focus during the experiment. For this reason, only 134 of the catalogued Rhod5F rcTLP-uncoating events had enough signal in the 561-nm channel for further analysis of the Ca^2+^ sensor (Fig. 10).

**FIG 11:**
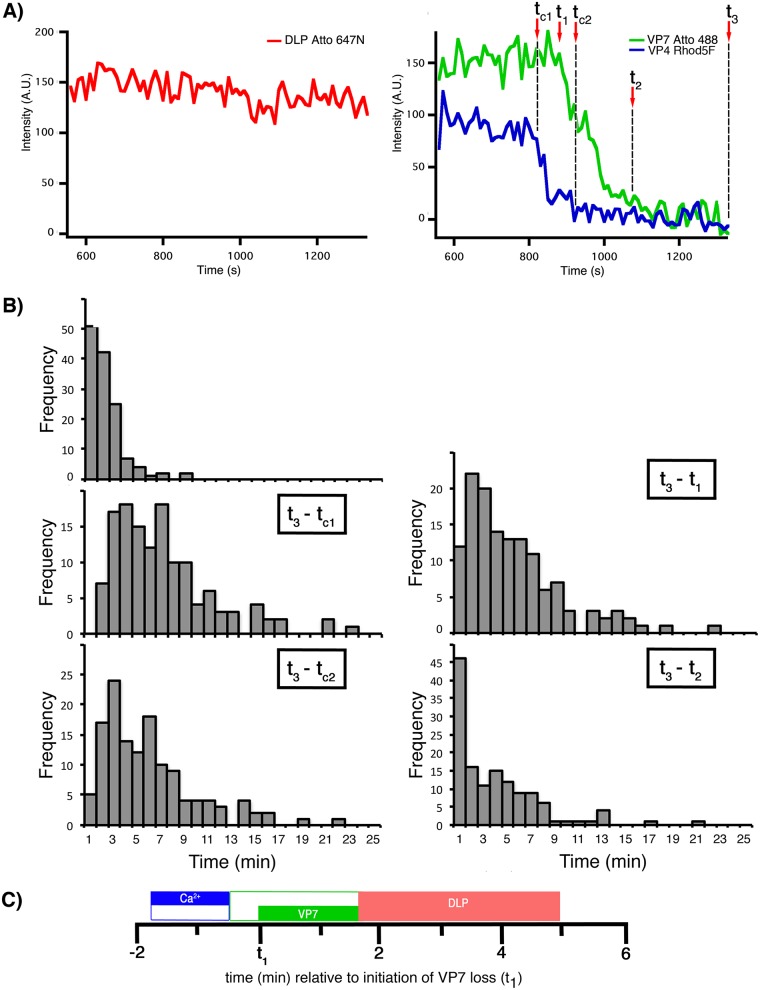
Kinetics of VP7 and Ca^2+^ loss and DLP release from Rhod5F rcTLPs. (A) Traces for DLP (left) and for VP7 and VP4 Rhod5F (right). Dashed vertical lines show the onset and termination of VP7 loss (*t*_1_ and *t*_2_, respectively), the onset and termination of Ca^2+^ loss (*t*_c1_ and *t*_c2_, respectively), and DLP release (*t*_3_). (B) Distribution of intervals between the onset of VP7 and Ca^2+^ loss (*t*_1_ − *t*_c1_), the onset of Ca^2+^ loss and DLP release (*t*_3_ − *t*_c1_), the onset of VP7 loss and DLP release (*t*_3_ − *t*_1_), termination of Ca^2+^ loss and DLP release (*t*_3_ − *t*_c2_), and termination of VP7 loss and DLP release (*t*_3_ − *t*_2_), from 134 uncoating particles collected from 35 BSC-1 cells infected at an MOI of 10. (C) Schematic of the average times outlined in panel B showing the timing and duration of VP7 loss (green bar), Ca^2+^ loss (blue bar), and overall DLP release (red bar) in relation to *t*_1_.

For these 134 uncoating events, we found that the loss of the Rhod5F signal (i.e., loss of Ca^2+^ from the vesicle surrounding the particle) began 1.8 ± 0.1 min before the initiation of VP7 signal loss (*t*_1_ − *t*_c1_) and 3.3 ± 0.2 min before the end of uncoating (*t*_2_ − *t*_c1_) (Fig. 11 and Table 4). The duration of the entire process, from the initiation of Ca^2+^ signal loss to DLP release (*t*_3_ − *t*_c1_), was 6.7 ± 0.4 min.

**TABLE 4:** Kinetics of VP7 and Ca^2+^ loss, and DLP release, from Rhod5F rcTLPs^*a*^

Interval	Avg time (min) ± SD	Median time (min)
*t*_1_ − *t*_c1_	1.8 ± 0.1	1.3
*t*_3_ − *t*_1_	5.0 ± 0.3	3.9
*t*_3_ − *t*_c1_	6.7 ± 0.4	5.8
*t*_3_ − *t*_2_	3.4 ± 0.3	2.3
*t*_3_ − *t*_c2_	5.5 ± 0.3	4.7

a Number of particles, 134; number of cells, 35.

## DISCUSSION

The results that we report here relate the timing of Ca^2+^ loss to other molecular events during rotavirus cell entry. In our previous work, we showed that an entering particle becomes inaccessible to the external medium within about 5 min of attachment and that release occurs 3 to 5 min later. We have now analyzed in detail the relationship between uncoating (loss of outer layer proteins VP7 and VP4) and release (abrupt onset of rapid diffusional motion of the uncoated DLP) while monitoring directly the leakage of Ca^2+^ from the vesicle surrounding an engulfed particle. The order of events is as follows: (i) sequestration of the virion from the external medium; (ii) a drop in Ca^2+^ concentrations to levels well below the *K_d_* (dissociation constant) of Rhod5F for Ca^2+^ (∼1 μM) and, hence, probably to the concentration of Ca^2+^ in the cytosol (∼0.01 μM); (iii) onset of VP7 and VP4 dissociation; (iv) complete loss of detectable outer layer proteins from the vicinity of the DLP; and (v) particle release. We discuss molecular mechanisms for each of these in turn.

(i) The RRV virion elicits the formation of an engulfing vesicle, which closes off about 5 min after initial attachment (12). Our previous work showed not only that penetration is pH independent but also that particles that reach the reduced-pH environment of early endosomes never uncoat. Our experiments confirm these results: pHrodo Red does not fluoresce at any point along the traces of particles that lose VP4 and VP7, while those particles that show pHrodo fluorescence do not subsequently shed their outer layers, presumably because of their localization in an early endosome. Given that the fluorescence of Rhod5F can be enhanced by the addition of protons near the Ca^2+^ binding site of the fluorophore at lower pHs, this control is important for confirmation that the Rhod5F signal coincident with an uncoating virion never experiences a decrease in pH. Therefore, the addition of a wavelength to correct for a potential pH-induced Rhod5F signal enhancement can be circumvented in this case, and the three available wavelengths on the spinning-disk confocal microscope can be used to monitor dynamics of Ca^2+^, VP7, and DLP.

Although impaired in infectivity, the Rhod5F particles are defective principally at the attachment step. When 10,000 Rhod5F particles/cell were added, we detected 78 associated with the cell after 1 h, of which 26 (33%) had uncoated. The corresponding numbers for unmodified 469C particles were 204 and 94 (46%). Both of these uncoating efficiencies are comparable to those that we have determined previously. Moreover, our recent work has shown that about 10% of uncoated particles become transcriptionally active, at a level detectable by fluorescence *in situ* hybridization, yielding MOI estimates of 2.6 and 9.4 for Rhod5F and wt particles, respectively. In view of the variation found previously (16), these numbers agree adequately with the directly determined MOIs of 1 and 20. Thus, once attached, Rhod5F and wt particles appear to have indistinguishable properties. The position of the bulky DBCO linker plus the Rhod5F sensor is close to the VP5*-VP8* interface; inefficient attachment is a reasonable consequence of its size and location.

(ii) We estimate from electron micrographs that the inner diameter of the engulfing vesicle is about ∼1,100 Å (12). Taking ∼850 Å as the mean diameter of the particle, there are about 500 free ions (assuming a 2 mM concentration of Ca^2+^) in the gap between the mean virion surface and the inner leaflet of the vesicular membrane. This number is smaller than the total number of virion-bound Ca^2+^ ions (1,560, assuming full occupancy) at the VP7 trimer interfaces, and the rate-limiting step for Ca^2+^ exit is probably dissociation from the virion itself. There may also be a pool of Ca^2+^ ions inside the virion, associated with the viral RNA. The relatively slow course of Ca^2+^ loss (comparable to the kinetics of subsequent VP7 loss) probably reflects the dissociation time(s) and not the conductance of the Ca^2+^ leak in the vesicular membrane.

(iii) Our results show that loss of Ca^2+^ always precedes loss of the outer layer proteins, an inference previously derived only from biochemical properties of purified virions. They also show that VP4 and VP7 leave the particle together, ruling out any contribution of VP4 dissociation to the loss of signal from Rhod5F. The Rhod5F signal reached baseline approximately 0.5 min before the apparent onset of VP7 dissociation. Thus, whatever compromise in membrane permeability that allows Ca^2+^ to pass from the vesicle into the cytosol is probably insufficient to allow proteins to leave, implying that Ca^2+^ loss induces some further, membrane-disrupting steps.

We have suggested previously that the interaction of the hydrophobic loops on VP5* with the vesicular membrane might create an initial Ca^2+^ leak, loosening the Ca^2+^-dependent VP7 trimer contacts and allowing VP5* to undergo a membrane-disrupting conformational rearrangement (12). This proposed sequence of molecular events is consistent with the order of fluorescence changes detected in the experiments reported here. Our ongoing structural studies of the VP5* conformational change on the virion surface promise to provide more-detailed, three-dimensional (3D) views of these conformational transitions.

(iv and v) Loss of the outer layer protein fluorescent signal in the diffraction-limited volume surrounding a DLP implies a disruption of the vesicle membrane extensive enough that relatively large proteins can diffuse away, but the mechanism of this disruption, by membrane coupling of the VP5* conformational change, awaits full elucidation. Equally puzzling is the delay between full uncoating and free diffusion of the uncoated DLP. The mean times between complete loss of VP7 and DLP release varied between 2.8 and 3.4 min for the various rcTLPs studied here. Indeed, even the vesicle-enclosed particle, while apparently fully pinched off from the plasma membrane, does not move rapidly away from the cell surface. Thus, the timing of full rupture of the surrounding vesicle is not necessarily defined by the release time of the DLP. Electron microscopy has not yet suggested a mechanism for this retention. If the vesicle or the initially liberated DLP were entrapped by cortical actin, we might expect that escape would require capture of a cytoskeletal motor, but our previous analysis of release showed a trajectory characteristic of free diffusion. New correlated light and electron microscopy studies may help resolve the puzzle.

Many other nonenveloped viruses appear to have evolved mechanisms for using a steep Ca^2+^ gradient from the outside to the inside of a cell. For example, in polyomavirus particles, both Ca^2+^ and disulfides lock the interactions between the pentameric assembly units (3, 4). Disulfide exchange in the endoplasmic reticulum, an intermediate compartment *en route* to nuclear entry, is thought to release the disulfide constraints to uncoating; the timing and significance of Ca^2+^ release have not yet been determined (24–26). During entry of adenoviruses, signal transduction from a Ca^2+^ “leak” at the cell surface or from endosomes may facilitate viral uptake and infection (27). The use of a Ca^2+^ sensor, such as Rhod5F, attached to the virion and hence reporting a relevant local Ca^2+^ concentration, might clarify the role of Ca^2+^ in controlling these entry-related processes.

## MATERIALS AND METHODS

### Cells.

Adherent Cercopithecus aethiops BSC-1 and MA104 cells (ATCC) were maintained at 37°C and 5% CO_2_. BSC-1 cells were grown in DMEM (Invitrogen Corporation) supplemented with 10% fetal bovine serum (FBS) and 1× GlutaMAX (Thermo Fisher Scientific). MA104 cells were similarly maintained in M199 medium with 10% FBS.

### Mutagenesis.

The VP4 serine 469-to-cysteine (469C) mutation was generated from the pfastBac-1 vector (Invitrogen) using mutagenic primers (IDT). The mutation was verified by sequencing (Dana-Farber/Harvard Cancer Center) through the VP4-encoding region after transformation into, and purification from, Escherichia coli DH5α.

### TLP, DLP, and recombinant protein purification.

TLPs, DLPs, VP7, and VP4 were purified as previously described (9). For TLP and DLP production, MA104 cells were grown in 850-cm^2^ roller bottles (Corning), and confluent monolayers were infected with rhesus rotavirus (RRV) (G3 serotype) at an MOI of 0.1 focus-forming units (FFU)/cell in M199 medium supplemented with 1 mg/ml porcine pancreatic trypsin (Worthington Biochemical). Cell culture medium was collected at 24 to 36 h postinfection, when cell adherence was <5%. TLPs and DLPs were purified by freeze-thawing, ultracentrifugation, Freon-113 extraction, and separation on a cesium chloride gradient. TLPs were desalted with a 5-ml Zeba spin column (Thermo Fisher) into a solution containing 20 mM Tris (pH 8.0), 100 mM NaCl, and 1 mM CaCl_2_ (TNC). DLPs were desalted into a solution containing 20 mM HEPES (pH 7.5) and 100 mM NaCl (HN). Infectivity of native virions was determined as previously described (22, 28), and specific infectivities were determined using concentrations based on densitometry measurements of an SDS-PAGE gel.

VP7 and VP4 were expressed in Sf9 cells infected with a baculovirus vector. VP7 was purified by successive affinity chromatography on concanavalin A and monoclonal antibody (mAb) 159, specific for the VP7 trimer (elution by EDTA). Purified VP7 was desalted into a solution containing 2 mM HEPES (pH 7.5), 10 mM NaCl, and 0.1 mM CaCl_2_ (0.1 HNC).

For VP4, harvested cells were lysed by freeze-thawing and clarified by centrifugation after the addition of a cOmplete EDTA-free protease inhibitor (Roche). VP4 was precipitated by the addition of ammonium sulfate to 30% saturation, pelleted, and resuspended in a solution containing 25 mM Tris (pH 8.0), 10 mM NaCl, and 1 mM EDTA (T10NE), which was matched to the conductance of Phenyl HP start buffer (25 mM Tris [pH 8.0], 3.5 M NaCl, 1 mM EDTA), and the solution was loaded onto a Phenyl HP column (GE Healthcare). Following elution with T10NE, fractions containing VP4 were pooled, dialyzed against the same buffer, loaded onto a HiTrap Q column (GE Healthcare), and eluted in Phenyl HP start buffer. Pooled fractions containing VP4 were then concentrated to 600 μl with a Centriprep 50 concentrator (Millipore) and subjected to a final purification on an S200 size exclusion column (GE Healthcare) in a solution containing 20 mM HEPES (pH 7.5), 100 mM NaCl, and 1 mM EDTA (HNE).

### Fluorescent labeling of DLP, VP7, and VP4.

50 μg DLP was brought to a volume of 100 μl in HN, to which 11.1 μl 1 M NaHCO_3_ (pH 8.3) was added. This solution was then added to 1.11 μl of 500 μg/ml Atto 647N NHS ester dye. The reaction proceeded for 1 h at room temperature before quenching with 12 μl of 1 M Tris (pH 8.0). The sample was then desalted through a 0.5-ml Zeba spin column into a solution containing 20 mM Tris (pH 8.0) and 100 mM NaCl (TN).

VP7 was brought to 1.08 mg/ml in a total volume of 75.5 μl using 0.1 HNC, and 8.4 μl of 1 M NaHCO_3_ (pH 8.3) was added. This solution was mixed into 0.84 μl of 340 μg/ml Atto 488 NHS ester. The reaction proceeded at room temperature for 1 h before quenching with 9 μl of 1 M Tris (pH 8.0). The labeled VP7 was then desalted into a solution containing 2 mM Tris, (pH 8.0), 10 mM NaCl, and 0.1 mM CaCl_2_ (0.1 TNC).

For Atto 565 labeling, wt VP4 was brought to 5.0 mg/ml in a total volume of 85.8 μl with HNE, and 9.6 μl of 1 M NaHCO_3_ (pH 8.3) was added. This solution was mixed with 0.7 μl of 10 mg/ml Atto 565 NHS ester. The reaction proceeded at room temperature for 1 h before quenching with 10 μl of 1 M Tris (pH 8.0). The labeled VP4 was then desalted into a solution containing 20 mM Tris (pH 8.0), 100 mM NaCl, and 1 mM EDTA (TNE). The degree of labeling of the protein was found to be 1 Atto 565 molecule to 1 VP4 molecule based on absorbances at 280 nm and 560 nm, using a 280-nm correction factor of 0.164 and an extinction coefficient of the label of 12,000 M^−1^ cm^−1^, as described by the manufacturer.

For pHrodo Red labeling, wt VP4 was brought to 5 mg/ml in a total volume of 85.8 μl with HNE, and 9.6 μl of 1 M NaHCO_3_ (pH 8.3) was added. This solution was mixed with 0.44 μl of 6.7 mg/ml pHrodo Red. The reaction proceeded at room temperature for 1 h before quenching with 10 μl of 1 M Tris (pH 8.0). The labeled VP4 was then desalted into TNE. The degree of labeling of the protein was found to be 1 pHrodo Red molecule to 2 VP4 molecules based on absorbances at 280 nm and 560 nm, using a 280-nm correction factor of 0.12 and an extinction coefficient of the label of 65,000 M^−1^ cm^−1^, as described by the manufacturer.

Rhodamine 5F (Rhod5F) azide labeling of monomeric VP4 469C began by reacting the protein with dibenzocyclooctyne-maleimide (DBCO-maleimide; Sigma). A total of 250 μl VP4 469C at 3.2 mg/ml in HNE was added to 4 μl of 4.9 mg/ml DBCO-maleimide dissolved in dimethyl sulfoxide (DMSO). The reaction was allowed to proceed at room temperature for 1 h before quenching with 1.25 μl of 1 M dithiothreitol (DTT) in HNE.

DBCO-labeled VP4 469C was then desalted into HNE and diluted to 1 mg/ml with the same buffer. This protein was then added to enough 1 mM Rhod5F dissolved in DMSO to achieve a final Rhod5F concentration of 57.5 μM (about a 5-fold excess of label over protein; roughly 5.7% DMSO in the final reaction mixture). The Rhod5F reaction was allowed to proceed at room temperature for 3 h, followed by overnight incubation at 4°C. The following day, the protein was desalted, using a 5-ml Zeba spin column, into HNE and concentrated with a 50-kDa-cutoff Microcon instrument (Millipore). Labeling of the protein achieved a ratio of 1 Rhod5F molecule to 1 VP4 469C molecule based on absorbances at 280 nm and 548 nm, using a 280-nm correction factor of 0.274 and an extinction coefficient of the label of 78,000 M^−1^ cm^−1^.

Labeling of VP4 469C on rcTLPs was performed after the recoating reaction and is outlined below.

### Sequential chymotrypsin-trypsin digestion of VP4 469C-Rhod5F.

Ten micrograms of VP4 469C-Rhod5F was brought to a final volume of 10 μl by the addition of a solution containing 20 mM Tris (pH 8.0), 100 mM NaCl, and 0.1 mM EDTA (TN0.1E). A total of 1.26 μl of 50 μg/ml tosyl-l-lysyl-chloromethane (TLCK)-treated chymotrypsin (Worthington Biochemical) in TNC was added, and the sample was incubated at 37°C for 30 min. A total of 1.16 μl of 50 μg/ml tosyl phenylalanyl chloromethyl ketone (TPCK)-treated trypsin (Worthington Biochemical) in TNC was then added, and the sample was incubated at room temperature for 1 h. The VP4 CT samples were then quenched by the addition of phenylmethylsulfonyl fluoride (PMSF) to a final concentration of 1 mM. Both samples were mixed with a reducing sample buffer containing a final concentration of 200 mM DTT and either boiled at 95°C for 3 min or incubated at room temperature for 3 min. A 5-to-20% gradient gel was then run with both the boiled and unboiled CT samples as well as 2.8 μg of undigested labeled protein for comparison.

### Preparation of fluorescently labeled rcTLPs.

Recoating of rcTLPs containing Atto 565-labeled wt VP4 was performed according to previously described protocols (22), using the labeled VP7 and DLP prepared as outlined above. Briefly, 1 M sodium acetate (pH 5.2) was added to a volume of Atto-labeled DLP resulting in a final concentration of 100 mM sodium acetate. Atto 565-labeled VP4 was then added to a final concentration of 0.9 mg/ml (∼33-fold excess), and the mixture was incubated at room temperature for 1 h. Labeled VP7 was then added in a 2.3-fold excess along with a final addition of sodium acetate and CaCl_2_ to maintain their respective concentrations of 100 mM and 1 mM. The mixture was incubated at room temperature for 1 h. Recoated particles were separated from excess labeled components by cesium chloride gradient centrifugation and desalted with a 5-ml Zeba spin column into TNC. Recoating of rcTLPs containing pHrodo Red-labeled wt VP4 performed according to the same protocol, with the particles being desalted with a 5-ml Zeba spin column into a solution containing 20 mM HEPES (pH 7.5), 100 mM NaCl, and 1 mM CaCl_2_ (HNC).

rcTLPs containing Rhod5F-labeled VP4 469C were generated by first recoating with unlabeled VP4 469C according to the same protocol as the one outlined above. The purified, unlabeled rcTLPs in TNC were added to DBCO-maleimide such that the final concentration of DBCO-maleimide was 9.5 μM, corresponding to a final DMSO volume equal to 1% of the total reaction mixture volume. The reaction was allowed to proceed at room temperature for 2 h, followed by desalting with a 5-ml Zeba column into TNC.

The DBCO-labeled rcTLPs were then added to a volume of 1 mM Rhod5F such that the final label concentration was 20 μM, corresponding to a final DMSO volume equal to 2% of the total reaction mixture volume. The reaction mixture was incubated at 4°C overnight, after which the sample was desalted using a 5-ml Zeba column into HNC.

Titers of recoating reaction mixtures were determined by infectious focus assays as previously described (22, 28), and specific infectivities were derived through concentration measurements based on densitometry of a Western blot using a primary antibody specific for VP6 (antibody 2B4; Santa Cruz Biotechnology).

For infectivity measurements in the presence of Baf A1, cells were incubated for 1 h at 37°C with unsupplemented DMEM containing 1% DMSO and 100 nM Baf A1. Control cells were similarly incubated in unsupplemented DMEM or DMEM with 1% DMSO for 1 h. pHrodo Red rcTLPs were then added in DMEM containing 100 nM Baf A1 and 1% DMSO, 1% DMSO, or only DMEM, and infection was allowed to proceed for 1 h at 37°C before processing as for the assays described above.

### 469C and Rhod5F rcTLP binding assay.

Number 1.5 round glass coverslips (25 mm; Warner Instruments) were sonicated for 30 min in 3 M NaOH, rinsed thoroughly in water, and sonicated again for 20 min in 70% ethanol (EtOH). Each coverslip was then placed into a well of a 6-well culture plate (Corning) and rinsed twice in phosphate-buffered saline (PBS), followed by two rinses in unsupplemented DMEM. BSC-1 cells were plated in these wells and allowed to grow overnight to 50% confluence in supplemented DMEM.

On the day of the experiment, the coverslips were washed twice with prewarmed DMEM and infected with a viral load of about 10,000 particles per cell, corresponding to an MOI of 20 for 469C rcTLPs and an MOI of 1 for Rhod5F rcTLPs. Infection proceeded for 1 h at 37°C. The cells were then washed three times in HNC and fixed for 10 min at room temperature in HNC with 4% paraformaldehyde. Samples were then washed three times for 5 min in HNC prior to visualization.

### Confocal imaging.

Fixed-cell images were acquired with a Mariana system (Intelligent Imaging Innovations, Denver, CO) based on a Zeiss Axio-Observer inverted microscope (Carl Zeiss Microimaging Inc., Thornwood, NY) equipped with a CSU-X1 spinning-disk confocal unit (Yokogawa Electric, Tokyo, Japan), a piezo-driven z-translation, and linear encoded *x* and *y* translations and controlled with SlideBook V6.0 (Intelligent Imaging Inc., Denver, CO). Excitation wavelengths were 488, 561, and 640 nm (lasers were obtained from Cobolt, Solna, Sweden); the emission filters were 525/50-, 607/36-, and 680-nm long-pass filters (Semrock, Rochester, NY). z-series covering the full volume of the cells were acquired with a z-step of 0.5 μm. All wavelengths were acquired with 100-ms exposure times at 100% laser power.

For live-cell imaging, BSC-1 cells were grown on 8-chambered number 1.5 cover glass slides (Cellvis) in a volume of 400 μl supplemented DMEM and allowed to grow overnight to 50% confluence. Prior to imaging, all 8 chambers were washed three times with 400 μl Fluorobrite DMEM (Invitrogen) containing 5% FBS and 25 mM HEPES (pH 7) (Invitrogen), and a final volume of 200 μl of this imaging medium was added to the wells. For Baf A1 experiments, this 200 μl of medium also contained 1% DMSO and 100 nM Baf A1, which was incubated for 1 h prior to the addition of virus. The slides were then mounted on a Nikon Ti motorized inverted microscope with a Perfect Focus system enclosed in an OkoLab cage microscope incubator at 37°C with 5% CO_2_.

After the addition of virus to a chamber of interest (MOI = 10 for Rhod5F-labeled rcTLPs; MOI = 50 for Atto 565- and pHrodo Red-labeled rcTLPs), live-cell images were acquired every 10 s with 200-ms (642 nm), 300-ms (488 nm and 562 nm for Atto 565 and Rhod5F), and 500-ms (562 nm for pHrodo Red) exposure times for 30 min. All images were collected using a 60×, 1.4-numerical-aperture (NA) objective with a Yokagawa CSU-X1 spinning-disk confocal microscope with Spectral Applied Research Borealis modification. Images were acquired with a Hamamatsu Flash4.0 V3 sCMOS camera (2-by-2 binning) controlled by MetaMorph software using the Spectral Applied Research LMM-5 laser merge module with solid-state lasers controlled by acousto-optic tunable filter (AOTF) at the following wavelengths: 488 nm (100 mW), 561 nm (100 mW), and 642 nm (101 mW).

pHrodo Red-labeled rcTLPs on glass were imaged in 200 μl Fluorobrite DMEM containing 5% FBS and 25 mM HEPES (pH 7) as single z-series over 15 z-planes with a 0.5-μm step size with the exposure times outlined above. Two hundred microliters of 1 M sodium acetate at pH 5.2 was then added, and particles were once again imaged.

### Image analysis.

**FIG 12:**
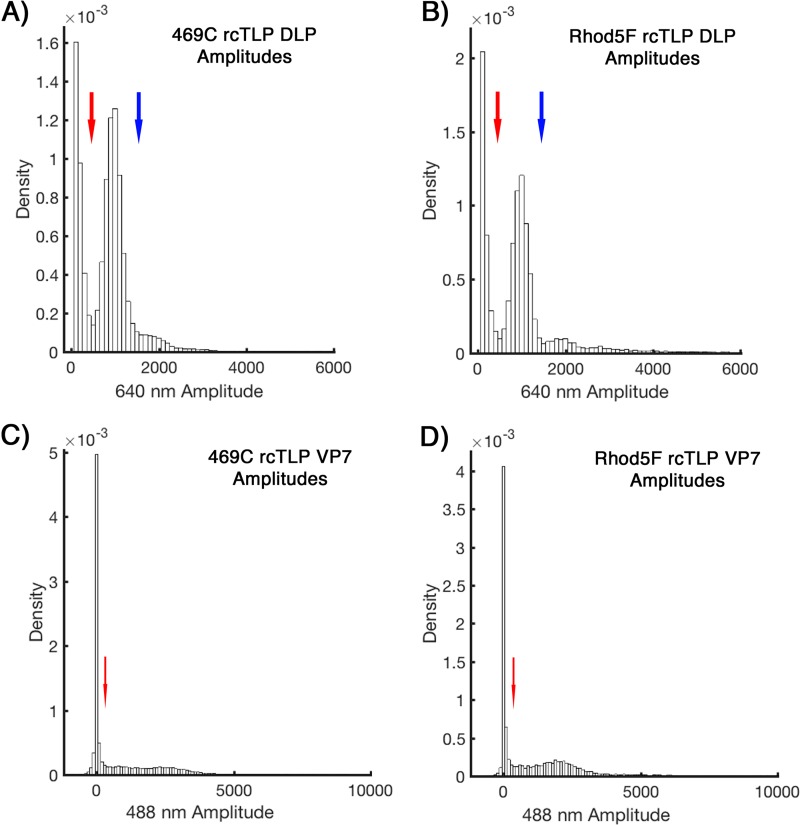
Probability densities of 640- and 488-nm channel amplitudes from 469C and Rhod5F rcTLP binding assays. (A and B) Distribution of 640-nm amplitudes derived from particle detections as described in Materials and Methods for all 469C (A) and Rhod5F (B) binding assay samples. Blue and red arrows indicate the upper and lower limits for detection to be considered a single particle; signals weaker than those indicated by the blue arrow were taken as noise, and signals stronger than those indicated by the red arrow were considered aggregates or multiple unresolved particles. (C and D) Distribution of 488-nm amplitudes derived from particle detections for all 469C (C) and Rhod5F (D) binding assay samples (see Materials and Methods). The red arrow points to the cutoff, above which a particle was taken to be a VP7-488-coated rcTLP and below which it was considered uncoated.

The signals from fluorescently labeled rcTLPs bound to fixed cells were detected by using custom MATLAB (MathWorks, Natick, MA) routines that fitted the amplitudes with a 3D Gaussian fitting function (29). Thresholds for a valid, single DLP/TLP were set between amplitudes of 450 and 1,550 for Atto 647N-labeled particles (Fig. 12A and B). Detections above these limits were considered aggregates or multiple unresolved particles. A particle was classified as a DLP, lacking VP7, if it had a 488-nm amplitude of >300 (Fig. 12C and D). Signals and tracking of fluorescently labeled rcTLPs in live-cell experiments were similarly processed using a two-dimensional (2D) version of the software (30).
